# Immunogenicity of Bioproducts: Cellular Models to Evaluate the Impact of Therapeutic Antibody Aggregates

**DOI:** 10.3389/fimmu.2020.00725

**Published:** 2020-05-05

**Authors:** Myriam Nabhan, Marc Pallardy, Isabelle Turbica

**Affiliations:** Inserm, Inflammation, Microbiome and Immunosurveillance, Université Paris-Saclay, Châtenay-Malabry, France

**Keywords:** immunogenicity, monoclonal antibodies, aggregation, danger signal, antigen, cell-based models

## Abstract

Patients treated with bioproducts (BPs) frequently develop anti-drug antibodies (ADAs) with potential neutralizing capacities leading to loss of clinical response or potential hypersensitivity reactions. Many factors can influence BP immunogenicity and could be related to the patient, the treatment, as well as to the product itself. Among these latter factors, it is now well accepted that BP aggregation is associated with an increased potential for immunogenicity, as aggregates seem to be correlated with ADA development. Moreover, the presence of high-affinity ADAs suggests a CD4 T-cell dependent adaptive immune response and therefore a pivotal role for antigen-presenting cells (APCs), such as dendritic cells (DCs). In this review, we address the *in vitro* methods developed to evaluate how monoclonal antibodies could trigger the immunization process by focusing on the role of aggregated antibodies in the establishment of this response. In particular, we will present the different cell-based assays that have been used to assess the potential of antibodies and their aggregates to modulate cellular mechanisms leading to activation and the biological parameters (cellular activation markers, proliferation and secreted molecules) that can be measured to evaluate the different cell activation stages and their consequences in the propagation of the immune response. Indeed, the use of such strategies could help evaluate the risk of BP immunogenicity and their role in mitigating this risk.

## Introduction

Bioproducts (BPs) such as recombinant proteins, including monoclonal antibodies, have proven to be effective in growing therapeutic areas and in particular for the treatment of chronic diseases. However, an ongoing concern while using these therapeutics is their immunogenicity, which results in the production of anti-drug antibodies (ADAs) in treated patients. ADAs detected in patients’ sera are mainly IgG1 and IgG4, although IgE and transient IgM have also been evidenced in patients developing hypersensitivity reactions (1, 2). Depending on the drug, ADA specificity is variable. For therapeutic antibodies, ADAs are mainly directed against epitopes recognized as foreign, e.g., mouse remaining epitopes in chimeric or humanized antibody paratopes, human allotopes, or human idiotopes (3). Moreover, some ADAs have a neutralizing activity, while others do not. For instance, in the case of anti-TNF antibodies, ADA characterization showed that 90% of anti-infliximab antibodies are neutralizing and more than 97% of anti-adalimumab antibodies are also neutralizing (4). Studies exploring peptide sequences targeted by these ADAs identified B-cell epitopes notably on infliximab and adalimumab variable regions, close to the paratope (5, 6). ADA development may lead to reduced BP serum concentrations due to the formation of immune complexes and a loss of efficacy (7) or adverse effects such as infusion reactions (8), cytokine release syndrome (9), or hypersensitivity reactions (2, 10). Immunogenicity is a growing issue that leads European and North American health authorities to regularly update recommendations in this area (11–13). Many factors can influence BP immunogenicity and they are related to the patient’s immunological and genetic status, the followed therapeutic regimen, as well as BP inherent characteristics and quality (14). The latter include the presence of aggregated BPs upon administration as an increased risk that promotes immunogenicity. In fact, aggregates have been correlated with ADA production (mainly IgG1 and less frequently IgG2a, IgG2b, IgG3, and IgM) in mouse models, either in wild type (15–19) or transgenic animals (20–23). In particular, it has been shown that the break of tolerance induced by antibody aggregates was dependent on the chemical modifications induced by the aggregation process (23). Moreover, it has been shown that T-cell help is required for ADA production in response to aggregates in transgenic and wild-type mice given that CD4 T-cell depletion abolished the immune response to aggregates (21). Clinical evidence of aggregates involvement in BP immunogenicity is rare and has only been described for human gamma globulin preparations and recombinant forms of endogenous proteins, such as human growth hormone, erythropoietin, or interferons [reviewed by Moussa et al. (24)]. While direct clinical evidence has not been reported, a number of review articles have dealt with the immunological mechanisms that could lead to ADA production (24–26). The predominance of high-affinity IgG as patients’ ADA main isotype suggests that ADA production arises from a T-cell-dependent immune response, in which BPs and/or aggregates could undergo APC uptake, to be processed and presented to T helper cells. In this context, the main professional APCs for peptide presentation are DCs that have a pivotal role in the efficient initiation of a specific immune response. It is therefore of high interest to focus on the mechanisms that underlie aggregates interactions with this innate/adaptive interface, to gain insight into BP immunogenicity.

In this review, we focus on cellular models that have been reported in the literature to assess the impact of aggregated monoclonal antibodies on the initiation of the immune response. As such, we will describe that antibody aggregates can behave as danger signals recognized by innate immune cells, but might also induce some alterations in the processing and presentation of antigens generated from the therapeutic antibody.

## Aggregation Process

The antibody aggregation process has been widely studied since the production of therapeutic antibodies at an industrial scale. Aggregation can occur at any stage of the manufacturing process, storage, transportation, or preparation for patient administration, under the influence of several critical environmental parameters (e.g., temperature, pH, ionic strength, shear forces, light, etc.). These stress conditions can alter the protein structure either by physical or by chemical damage and trigger the protein aggregation through different pathways. Thus, aggregation can occur as a result of the interaction of two monomers, either folded or unfolded, to gradually form reversible oligomers and then the initial irreversible aggregation nucleus, that is the starting point of aggregate growth. For monoclonal antibodies, the exposure of hydrophobic sequences representing the aggregation-prone regions (APRs) can promote oligomerization (27); these APRs are notably found in the complementarity-determining regions (CDRs) (28, 29) but can also be found in other positions on variable and constant regions of the antibodies (30). On the other hand, potential monomer self-association regions in Fab domains have also been identified (31). Moreover, a variety of high molecular complexes were evidenced when antibodies interact with their soluble target (32). Aggregation mechanisms are extensively described in two reviews (30, 33). Aggregates are usually described according to several criteria (size, reversibility, conformation, and shape), although the size has been adopted as the most convenient to suggest a classification (34), summarized in Table 1. Interestingly, the type of generated aggregates depends on the nature of the applied stress as well as the chosen monoclonal antibody. In particular, aggregated antibody preparations under accelerated experimental stresses induce a wide variety of aggregates. A classification scheme was proposed for antibody aggregates, based on several biophysical characterizations and the visible or subvisible criteria (35). Nevertheless, a few reports dealt with the occurrence of antibody aggregation that could take place during or just before the administration of the BP (36). For example, dilution of antibody preparations in PBS or in the manufacturer’s formulation led to a reduction in the monomer concentration (37). Moreover, nanometer, submicron, and micron protein particles have been evidenced in intravenous saline bags (38, 39). Furthermore, micron aggregates were detected in ejected solutions from prefillable syringes due to increased antibody adsorption to the syringe surface (40). Interestingly, it was shown that bedside filtration could significantly reduce the quantity of submicron- and micron-sized aggregates before BP injection (41). Finally, it is suggested that subcutaneous administration of antibody solutions could also favor aggregation due to the forced interaction between monomers in highly concentrated solutions (42, 43).

**TABLE 1:** Classification of protein aggregates (34).

**Size**	**Preferred terminology**	**Characteristics**
<100 nm	Nanometer aggregated	Oligomers, soluble
100–1000 nm	Submicron aggregates	Soluble
1–100 μm	Micron aggregates	Subvisible particles, insoluble
>100 μm	Aggregates greater than 100 μm	Visible particles, insoluble

## Antibody Aggregates Act as Danger Signals for Innate Immune Cells

*In vitro* cell-based models are valuable tools in an attempt to describe the interaction of exogenous molecules with the immune system. Monocytes and DCs are professional APCs acting as sensors while continuously capturing exogenous molecules that could represent a potential danger. The so-called “danger signal” concept (44) includes exogenous pathogen-associated molecular patterns (PAMPs), endogenous damage-associated molecular patterns (DAMPs), and the more recently described nanoparticle-associated molecular patterns (NAMPs) (45). Soluble submicron-sized protein aggregates can fall into the latter category (46). These molecular patterns can bind to pattern recognition receptors (PRR) expressed on innate immune cells and induce cell activation, through the activation of signaling pathways that lead to the activation of transcription factors such as nuclear factor kappa B (NF-kB) and activator protein 1 (AP-1), resulting in the secretion of pro-inflammatory cytokines and chemokines.

### Interaction of Antibody Aggregates With Peripheral Blood Mononuclear Cells

As a first approach, most cellular models that evidenced the danger signal role of antibody aggregates prepared under accelerated conditions used PBMCs from healthy donors. Joubert et al. first described a cytokine/chemokine signature resulting from PBMC activation in response to stir-stressed antibody preparations, compared to monomeric antibodies (47). Furthermore, using the same cellular model, a comparison of size-fractionated aggregates showed that aggregates having a size between 5 and 10 μm were the most efficient to induce cytokine secretion (48). PBMC activation was also induced by aggregated polyvalent immunoglobulin preparations (IVIG) in terms of cytokine and chemokine secretion (49, 50), but also in terms of intracellular proteins involved in signaling pathways: the activation of mitogen-activated protein kinases (MAPKs) p38, Erk1/2, and Jnk, was observed within 30 min of PBMC stimulation with IVIG aggregates. Screening of the expression of over 100 genes in PBMCs in response to aggregated IVIG showed an increased expression of specific genes implicated in cell signaling and/or linked to the activation and recruitment of innate immune cells (50).

Attempts to identify cellular receptors implicated in PBMC activation gave different outcomes, depending on the used cell-based model. Using specific blocking antibodies, the involvement of the toll-like receptors (TLRs), TLR2 and TLR4, and to a lesser extent the Fc-fragment receptors FcγRI and FcγRIII was evidenced via the decrease of aggregate-induced PBMC cytokine and chemokine secretion (47, 49). However, studies using reporter cell models allowing the evaluation of the individual implication of TLRs and/or FcγRs showed opposing results. Indeed, Polumuri et al. showed no implication of TLRs, including TLR2 and TLR4, in the activation of HEK293 cells expressing TLRs in response to IVIG aggregates (50). More recently, the use of other reporter cell models showed that antibody aggregates induced FcγRs activation, mainly FcγRIIa and FcγRIIIa; however, they did not activate TLRs (51). Taken together, all these results indicate that the activation of PBMCs in response to aggregated antibodies is multifactorial, through the potential engagement of multiple receptors. The implication of multiple cell types in the observed response of PBMCs clearly shows the complexity of this cellular model to evaluate the specific pathways (mainly receptors and intracellular proteins) enabled by antibody aggregates. It would therefore be of interest to study the role of each cell type separately to deepen our understanding of the innate immune response to aggregates.

### Interaction of Antibody Aggregates With Antigen-Presenting Cells

Aggregated antibody behavior as a danger signal was also demonstrated using APC models. The monocytic THP-1 cell line was compared to primary purified monocyte preparations to evaluate the impact of aggregated IVIG. Both pro-inflammatory cytokine secretion profiles were comparable, not in magnitude, but in terms of a dose-dependent response to increasing aggregates concentrations (49). Moreover, receptors involved in THP-1 or monocyte activation in response to aggregates were identified as TLR2, TLR4, and, to a lesser extent, FcγRII. Other studies focused on the impact of aggregated antibodies on DC maturation, using purified monocyte preparations from healthy donors that were differentiated into dendritic cells (moDCs). This primary cell *in vitro* model has been described and used for DC maturation studies under the action of coagulation Factor VIII preparations (52) and recombinant human growth hormone aggregates (53). The effect of monoclonal antibody aggregates on moDC has also been studied with rituximab (53–55), trastuzumab (54), monoclonal IgG1s (56), and adalimumab, natalizumab, and infliximab (55). These studies showed that antibodies in their native state did not induce maturation of moDCs whereas their aggregated counterparts increased the expression of surface markers, mainly CD83 and CD86, as well as the secretion of cytokines such as IL-6, IL-8, IL-10, IL-12p40, and chemokines such as CCL2, CCL3, and CCL4. Although moDC phenotypic alterations could be observed in response to different antibody aggregates, the degree of maturation varied depending on the used therapeutic antibody as well as the size, structure, quantity, and type of generated aggregates. For instance, Morgan et al. compared the moDC response to infliximab and natalizumab aggregates. This study showed that infliximab had the highest propensity to aggregate when submitted to heat stress contrary to natalizumab. Evaluating moDC maturation then showed that heat-stressed infliximab induced phenotypic alterations of moDCs whereas stressed natalizumab did not have any effect on moDC activation (55). Moreover, it was shown that infliximab aggregates increased the phosphorylation of the kinases Syk, Erk, and Akt (55).

**FIGURE 1:**
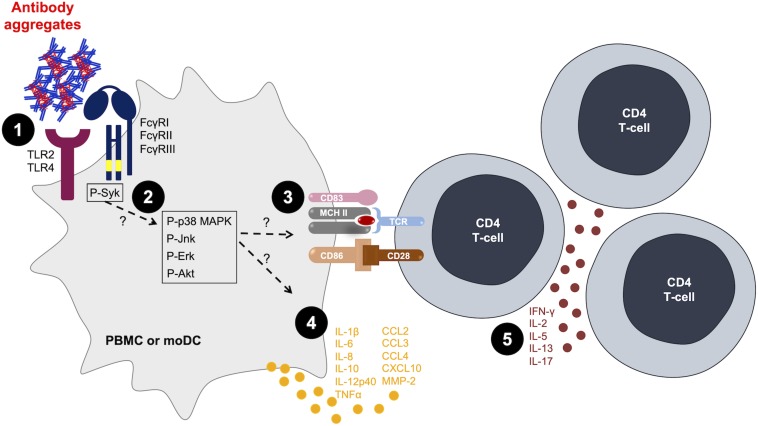
Antibody aggregates act as danger signals for innate immune cells. [1] Aggregates interact with antigen-presenting cells (APCs) via Fcγ receptors (FcγRs) or toll-like receptors (TLRs) (47, 49–51). [2] Aggregates induce the phosphorylation of intracellular signaling kinases (50, 55). [3; 4] Aggregates induce the activation of APCs in terms of [3] increased surface markers expression (53, 55, 56) and [4] increased cytokine and chemokine production (47, 53, 55). [5] The activation of APCs is sufficient to increase T-cell proliferation and cytokine production (53, 57). MoDC, monocyte-derived dendritic cells; PBMC, peripheral blood mononuclear cells.

In order to correlate the innate immune response observed with aggregated antibodies with the adaptive immune response, T-cell proliferation and cytokine secretion have been evaluated in multiple studies through PBMC culture models or allogeneic DC–T-cell co-culture models. On one hand, cultures of healthy donors’ PBMCs showed an increased proliferation of T cells and IL-2 production (57, 58) or IFN-γ production (56) in response to aggregated antibodies, whereas native antibodies induced little to no T-cell response. On the other hand, using an allogeneic moDC–T-cell co-culture model, we showed an increase in CD4 T-cell activation (proliferation and cytokine secretion) when moDC were treated with various types of protein aggregates [stir-stressed rituximab and stir-stressed recombinant human growth hormone (53)]. Interestingly, these aggregates did not induce similar cytokine profiles: rituximab aggregates induced IFN-γ, IL-5, IL-13, and IL-17 production, whereas growth hormone aggregates only induced IFN-γ secretion (53). These results strongly suggest that different mechanisms are implicated in the activation of moDCs depending on the origin and type of the generated protein aggregates. However, the results converge to show full DC activation, sufficient to trigger a T-cell response evaluated using orthogonal readouts such as proliferation and cytokine secretion (Figure 1). This suggests that antibody aggregates have the ability to initiate an adaptive immune response that could lead to ADA production.

## Antibody Aggregates and the Generation of *De Novo* Antigens

Producing high-affinity ADAs against therapeutic antibodies requires that patients develop a specific adaptive immune response through antigen recognition by T cells and B cells. In fact, specific T-cell activation requires efficient antigen presentation by fully mature DCs, through the establishment of the immune synapse (59, 60) that allows the transmission of three activation signals. The interaction between DCs and T cells is established via the HLA–peptide complex recognized by a specific T-cell receptor (TCR), the membrane co-stimulation proteins as well as the DC-secreted pro-inflammatory cytokines that allow T-cell proliferation and polarization (59, 61). Cell-based models presented in the previous section highlighted that aggregated antibodies could fulfill the two latter activation signals. However, the role of aggregates in the initiation of a specific T-cell response has been less explored (62), even though the specificity of the immune response to native monoclonal antibodies has been well documented over the past years, as described hereafter.

### The CD4 T-Cell Repertoire Targeting Monoclonal Antibodies

**FIGURE 2:**
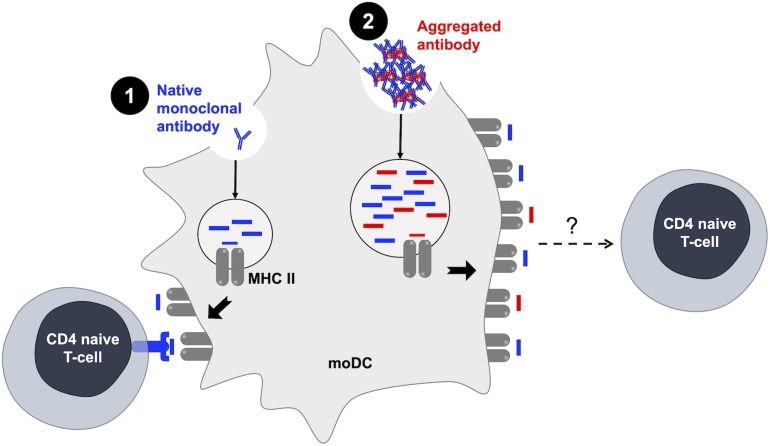
Antibody aggregates and the generation of *de novo* antigens. [1] The pre-existence of CD4 T-cell repertoires recognizing therapeutic monoclonal antibodies is evidenced in healthy donors (63). [2] Aggregates induce an increase in the number and the variety of MHC II-presented peptides (56). MHC II, major histocompatibility complex; moDC, monocyte-derived dendritic cells.

In order to establish a link between the development of the adaptive immune response and the clinically observed immunogenicity of monoclonal antibodies, autologous DC–T-cell co-culture models have been developed. Briefly, CD4 T cells isolated from human healthy donors’ blood are seeded in plates and stimulated by autologous mature moDCs loaded with monoclonal antibodies. After weekly rounds of stimulations with antibody-treated moDCs allowing specific T-cell expansion, an IFN-γ ELISpot assay is used to detect antibody-specific T-cell lines (CD4 T cells present in a single well) and determine a frequency of CD4 T cells recognizing monoclonal antibodies following the Poisson distribution for rare events. This cellular model first allowed Delluc et al. to identify the existence of T-cell repertoires recognizing monoclonal antibodies, notably rituximab, infliximab, and adalimumab. Interestingly, these results correlated with the clinical immunogenicity of BPs (63). More recently, studies helped identify the T-cell epitopes incriminated in monoclonal antibody immunogenicity, which is essential in the process of deimmunizing antibodies. Monoclonal antibodies can be uptaken and processed by APCs such as DCs and the derived-linear peptides will be presented to T cells on major histocompatibility complex (MHC) molecules. These peptides could then be identified through an MHC-Associated Peptide Proteomics (MAPPs) assay. Identified peptides are then tested in the co-culture model to determine the ones that can induce a T-cell response, which depends on the peptide’s affinity to bind to MHC molecules and the recognition of the MHC–peptide complex by a specific TCR. This type of experiment has evaluated T-cell epitopes of different monoclonal antibodies such as infliximab, rituximab (64), natalizumab, adalimumab (65), secukinumab, and ixekizumab (66). Results showed that peptides inducing a CD4 T-cell response to a monoclonal antibody were mainly sequences deriving from CDRs and framework regions (FR) of the antibody variable domains. These studies clearly showed the pre-existence of CD4 T cells specific of peptides deriving from therapeutic antibodies which could favor their immunogenicity (Figure 2).

### The Role of Antibody Aggregates in the Initiation of a Specific Immune Response

While studying the role of aggregates in the activation of DCs, it has been shown that aggregated antibodies can be internalized by these APCs (54). Different mechanisms can be involved in the uptake of particles: phagocytosis, macropinocytosis, or clathrin-mediated endocytosis. In particular, aggregated particles of size larger than 0.5 μm are internalized by phagocytosis or macropinocytosis, whereas smaller particles are internalized by clathrin-mediated endocytosis (67). Studies exploring the role of antibody aggregates mainly generate these aggregates by submitting antibody solutions to extreme and accelerated stress conditions that often induced the formation of submicron- and micron-sized aggregates. This strongly suggests that these aggregates would be internalized by either phagocytosis or macropinocytosis; however, no data has yet confirmed this hypothesis. On the other hand, *in vivo* studies are currently focusing on the oligomeric antibodies and their immunogenicity (68, 69). Indeed, nanometric-sized aggregates could be found in preparations and are hardly ever detected and eliminated before the administration of the therapeutic antibody (38, 41). Contrary to subvisible aggregates, oligomers would probably be uptaken by endocytosis, which is yet to be confirmed (67). Further investigations are necessary to gain insight into the mechanisms of aggregate binding and trafficking into APCs.

Once internalized, particles are trafficked to the endocytic compartment to be processed and generated peptides are then loaded on MHC molecules. One study by Ahmadi et al. showed that rituximab aggregates were uptaken by moDCs and co-localized with HLA-DR molecules after 30 min of incubation (54). Moreover, Rombach-Riegraf et al. used the MAPPs assay to evaluate the peptides presented on MHC-II molecules from moDCs loaded with native or aggregated antibodies. This study showed that the aggregation of IgG monoclonal antibodies can induce an increase in the number as well as in the diversity of the peptides presented by MHC-II molecules compared to native monoclonal antibodies (56). This observation suggests that aggregation could induce alterations in the uptake and processing mechanisms of the antibody leading to changes in the peptides presented to CD4 T cells (Figure 2).

How could aggregation modulate the specific T-cell response detected for native antibodies? One study exploring the T-cell response to erythropoietin and heat- or tungsten-induced erythropoietin aggregates in an autologous moDC–T-cell co-culture model that aggregates induced an increase in T-cell proliferation compared to native erythropoietin (62). This is the only study to date evaluating the specific T-cell activation in response to BP aggregates. For antibodies, what is currently known about aggregation and T-cell epitopes is that the majority of hydrophobic APRs are found in the T-cell epitope sequences of therapeutic antibodies (70). More studies could help better understand if antibody aggregates could initiate a specific immune response. In particular, it remains to be clarified if aggregation favors the presentation of *de novo* T-cell epitopes that could be generated by possible alterations in protein cleavage, occurring at different sites compared with the monomeric antibody.

## Summary and Conclusion

**FIGURE 3:**
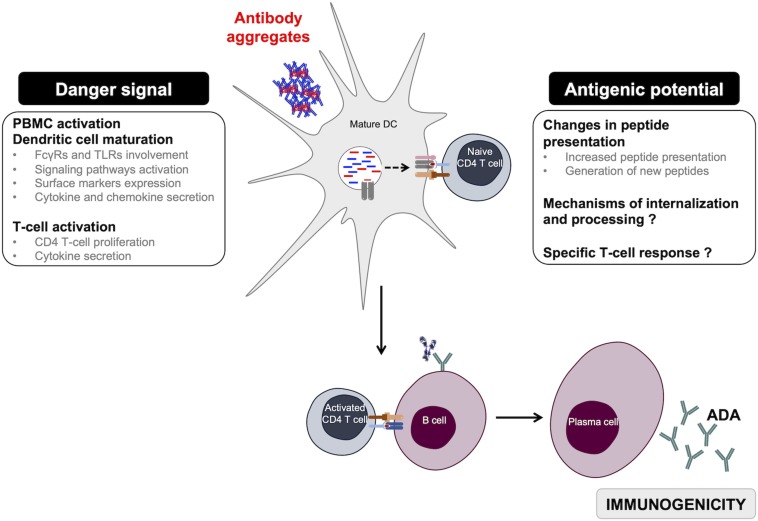
Overview of monoclonal antibody aggregates role in immunogenicity. Aggregated antibodies can have two complementary roles when in contact with APCs. They act as danger signals but also induce changes in antibody-peptide presentation, thus favoring the initiation of a specific T-dependent adaptive immune response driving anti-drug antibody development. ADA, anti-drug antibodies; DC, dendritic cells.

Immunogenicity of therapeutic monoclonal antibody aggregates has been widely explored in the past few years. In this review, we focused on the *in vitro* cellular models that have been used to better understand the role of aggregates in the initiation of a T-dependent immune response leading to the production of high-affinity ADAs (Figure 3). Many experimental studies have explored the danger signal role of antibody aggregates by showing their potential to induce efficient activation of innate immune cells. However, current data show the implication of diverse receptors, signaling pathways, surface markers, as well as cytokines and chemokines without a clear signature for all antibody aggregates. Exploring the differences between existing results show that the detected immune response can vary depending on the cellular model, the nature of the monoclonal antibody, the types of generated aggregates, as well as the level of the selected stress. It is important to notice that most studies have used extreme stress conditions that would often lead to the formation of aggregates of various size ranges, quantities, and structures that do not resemble aggregates found in administered preparations. Thus, it is currently essential to focus on the role of antibody oligomers in the initiation of the immune response; oligomers being sometimes detected but not efficiently eliminated from preparations. Using homogeneous well-characterized oligomer preparations may allow one to evaluate the sensitivity of these cellular models and to also determine a threshold of particle number sufficient for cell activation. Finally, the specificity of the immune response induced by antibody aggregates has yet to be explored to gain insight into the antigenicity of aggregates.

The use of cell-based assays has clearly some benefits in assessing the impact of aggregated antibodies on the establishment of the immune response. They allow one to directly work with a relevant mixture of human immune cells, and therefore to take into account the HLA diversity of donors. They also allow one to test a variety of samples and to compare different aggregate preparations. For these reasons, beyond their use for the assessment of induced cellular mechanisms, they can also find applications in the evaluation of BPs under development, to assess the potential risk of immunogenicity due to aggregation during the manufacturing process.

## Author Contributions

MN and IT wrote the manuscript. All authors contributed to manuscript revision, read, and approved the submitted version.

## Conflict of Interest

The authors declare that the research was conducted in the absence of any commercial or financial relationships that could be construed as a potential conflict of interest.

## Abbreviations

ADA: anti-drug antibody
APCs: antigen-presenting cell
BP: bioproduct
DC: dendritic cell
FcR: Fc receptor
IVIG: intravenous immunoglobulin G
MHC: major histocompatibility complex
moDC: monocyte-derived dendritic cell
PBMC: peripheral blood mononuclear cell
TLR: toll-like receptor.

## References

[1] van Schouwenburg PA Rispens T Wolbink GJ. Immunogenicity of anti-TNF biologic therapies for rheumatoid arthritis. *Nat Rev Rheumatol.* (2013) 9:164–72. 10.1038/nrrheum.2013.4 23399692

[2] Vultaggio A Matucci A Nencini F Pratesi S Parronchi P Rossi O Anti-infliximab IgE and non-IgE antibodies and induction of infusion-related severe anaphylactic reactions. *Allergy.* (2010) 65:657–61. 10.1111/j.1398-9995.2009.02280.x 19951375

[3] Bendtzen K. Immunogenicity of Anti-TNF-α Biotherapies: II. Clinical relevance of methods used for anti-drug antibody detection. *Front Immunol.* (2015) 6:109. 10.3389/fimmu.2015.00109 25904911 PMC4389574

[4] van Schie KA Hart MH de Groot ER Kruithof S Aarden LA Wolbink GJ The antibody response against human and chimeric anti-TNF therapeutic antibodies primarily targets the TNF binding region. *Ann Rheum Dis.* (2015) 74:311–4. 10.1136/annrheumdis-2014-206237 25342759

[5] Homann A Röckendorf N Kromminga A Frey A Jappe U. B cell epitopes on infliximab identified by oligopeptide microarray with unprocessed patient sera. *J Transl Med.* (2015) 13:339. 10.1186/s12967-015-0706-7 26511203 PMC4625721

[6] Homann A Röckendorf N Kromminga A Frey A Platts-Mills TA Jappe U. Glycan and peptide IgE epitopes of the TNF-alpha blockers infliximab and adalimumab – precision diagnostics by cross-reactivity immune profiling of patient sera. *Theranostics.* (2017) 7:4699–709. 10.7150/thno.20654 29187897 PMC5706093

[7] Papamichael K Vogelzang EH Lambert J Wolbink G Cheifetz AS. Therapeutic drug monitoring with biologic agents in immune mediated inflammatory diseases. *Expert Rev Clin Immunol.* (2019) 15:837–48. 10.1080/1744666X.2019.1630273 31180729

[8] Choquette D Faraawi R Chow A Rodrigues J Bensen WJ Nantel F. Incidence and management of infusion reactions to infliximab in a prospective real-world community registry. *J Rheumatol.* (2015) 42:1105–11. 10.3899/jrheum.140538 26077415

[9] Eastwood D Findlay L Poole S Bird C Wadhwa M Moore M Monoclonal antibody TGN1412 trial failure explained by species differences in CD28 expression on CD4+ effector memory T-cells. *Br J Pharmacol.* (2010) 161:512–26. 10.1111/j.1476-5381.2010.00922.x 20880392 PMC2990151

[10] Vultaggio A Matucci A Nencini F Pratesi S Petroni G Cammelli D Drug-specific Th2 cells and IgE antibodies in a patient with anaphylaxis to rituximab. *Int Arch Allergy Immunol.* (2012) 159:321–6. 10.1159/000336839 22846615

[11] European Medicines Agency *Guideline on Immunogenicity Assessment of Therapeutic Proteins: (EMEA/CHMP/BMWP/14327/2006 Rev 1).* Amsterdam: European Medicines Agency (2017). 24 p. 10.1159/000336839

[12] Kurki P. Compatibility of immunogenicity guidance by the EMA and the US FDA. *Bioanalysis.* (2019) 11:1619–29. 10.4155/bio-2018-0243 30672313

[13] U.S. Food and Drug Administration *Immunogenicity Assessment for Therapeutic Protein Products.* U.S. Food and Drug Administration (2014). Available online at: http://www.fda.gov/regulatory-information/search-fda-guidance-documents/immunogenicity-assessment-therapeutic-protein-products (accessed Jul 26, 2019).

[14] Dingman R Balu-Iyer SV. Immunogenicity of protein pharmaceuticals. *JPharmSci.* (2019) 108:1637–54. 10.1016/j.xphs.2018.12.014 PMC6720129 30599169

[15] Kijanka G Bee JS Korman SA Wu Y Roskos LK Schenerman MA Submicron size particles of a murine monoclonal antibody are more immunogenic than soluble oligomers or micron size particles upon subcutaneous administration in mice. *J Pharm Sci.* (2018) 107:2847–59. 10.1016/j.xphs.2018.06.029 30003898

[16] Kijanka G Bee JS Bishop SM Que I Löwik C Jiskoot W. Fate of multimeric oligomers, submicron, and micron size aggregates of monoclonal antibodies upon subcutaneous injection in mice. *J Pharm Sci.* (2016) 105:1693–704. 10.1016/j.xphs.2016.02.034 27044942

[17] Uchino T Miyazaki Y Yamazaki T Kagawa Y. Immunogenicity of protein aggregates of a monoclonal antibody generated by forced shaking stress with siliconized and nonsiliconized syringes in BALB/c mice. *J Pharm Pharmacol.* (2017) 69:1341–51. 10.1111/jphp.12765 28639328

[18] Shomali M Tanriverdi S Freitag AJ Engert J Winter G Siedler M Dose levels in particulate-containing formulations impact anti-drug antibody responses to murine monoclonal antibody in mice. *J Pharm Sci.* (2015) 104:1610–21. 10.1002/jps.24413 25737325

[19] Freitag AJ Shomali M Michalakis S Biel M Siedler M Kaymakcalan Z Investigation of the immunogenicity of different types of aggregates of a murine monoclonal antibody in mice. *Pharm Res.* (2014) 32:430–44. 10.1007/s11095-014-1472-6 25123991

[20] Haji Abdolvahab M Fazeli A Halim A Sediq AS Fazeli MR Schellekens H. Immunogenicity of recombinant human interferon beta-1b in immune-tolerant transgenic mice corresponds with the biophysical characteristics of aggregates. *J Interferon Cytokine Res.* (2016) 36:247–57. 10.1089/jir.2015.0108 26835734

[21] Bessa J Boeckle S Beck H Buckel T Schlicht S Ebeling M The immunogenicity of antibody aggregates in a novel transgenic mouse model. *Pharm Res.* (2015) 32:2344–59. 10.1007/s11095-015-1627-0 25630815

[22] Pisal DS Kosloski MP Middaugh CR Bankert RB Balu−iyer SV. Native-like aggregates of factor VIII are immunogenic in von Willebrand factor deficient and hemophilia a mice. *J Pharm Sci.* (2012) 101:2055–65. 10.1002/jps.23091 22388918 PMC3774159

[23] Boll B Bessa J Folzer E Ríos Quiroz A Schmidt R Bulau P Extensive chemical modifications in the primary protein structure of IgG1 subvisible particles are necessary for breaking immune tolerance. *Mol Pharm.* (2017) 14:1292–9. 10.1021/acs.molpharmaceut.6b00816 28206769

[24] Moussa EM Panchal JP Moorthy BS Blum JS Joubert MK Narhi LO Immunogenicity of therapeutic protein aggregates. *J Pharm Sci.* (2016) 105:417–30. 10.1016/j.xphs.2015.11.002 26869409

[25] Kraus T Winter G Engert J. Test models for the evaluation of immunogenicity of protein aggregates. *Int J Pharm.* (2019) 559:192–200. 10.1016/j.ijpharm.2019.01.015 30665000

[26] Ratanji KD Derrick JP Dearman RJ Kimber I. Immunogenicity of therapeutic proteins: influence of aggregation. *J Immunotoxicol.* (2014) 11:99–109. 10.3109/1547691X.2013.821564 23919460 PMC4002659

[27] Wang X Das TK Singh SK Kumar S. Potential aggregation prone regions in biotherapeutics: a survey of commercial monoclonal antibodies. *MAbs.* (2009) 1:254–67. 10.4161/mabs.1.3.8035 20065649 PMC2726584

[28] Perchiacca JM Bhattacharya M Tessier PM. Mutational analysis of domain antibodies reveals aggregation hotspots within and near the complementarity determining regions. *Proteins.* (2011) 79:2637–47. 10.1002/prot.23085 21732420

[29] Wang W Nema S Teagarden D. Protein aggregation—pathways and influencing factors. *Int J Pharm.* (2010) 390:89–99. 10.1016/j.ijpharm.2010.02.025 20188160

[30] Wang W Roberts CJ. Protein aggregation – mechanisms, detection, and control. *Int J Pharm.* (2018) 550:251–68. 10.1016/j.ijpharm.2018.08.043 30145245

[31] Lerch TF Sharpe P Mayclin SJ Edwards TE Lee E Conlon HD Infliximab crystal structures reveal insights into self-association. *MAbs.* (2017) 9:874–83. 10.1080/19420862.2017.1320463 28421849 PMC5524157

[32] Kohno T Tam L-TT Stevens SR Louie JS. Binding characteristics of tumor necrosis factor receptor-Fc fusion proteins vs anti-tumor necrosis factor mAbs. *J Invest Dermatol Symp Proc.* (2007) 12:5–8. 10.1038/sj.jidsymp.5650034 17502862

[33] Vázquez-Rey M Lang DA. Aggregates in monoclonal antibody manufacturing processes. *Biotechnol Bioeng.* (2011) 108:1494–508. 10.1002/bit.23155 21480193

[34] Narhi LO Schmit J Bechtold-Peters K Sharma D. Classification of protein aggregates. *J Pharm Sci.* (2012) 101:493–8. 10.1002/jps.22790 21989781

[35] Joubert MK Luo Q Nashed-Samuel Y Wypych J Narhi LO. Classification and characterization of therapeutic antibody aggregates. *J Biol Chem.* (2011) 286:25118–33. 10.1074/jbc.M110.160457 21454532 PMC3137085

[36] Nejadnik MR Randolph TW Volkin DB Schöneich C Carpenter JF Crommelin DJA Postproduction handling and administration of protein pharmaceuticals and potential instability issues. *J Pharm Sci.* (2018) 107:2013–9. 10.1016/j.xphs.2018.04.005 29665382

[37] Giannos SA Kraft ER Zhao Z-Y Merkley KH Cai J. Formulation stabilization and disaggregation of bevacizumab, ranibizumab and aflibercept in dilute solutions. *Pharm Res.* (2018) 35:78. 10.1007/s11095-018-2368-7 29492680 PMC5830485

[38] Pardeshi NN Qi W Dahl K Caplan L Carpenter JF. Micro- and nanoparticles delivered in intravenous saline and in an intravenous solution of a therapeutic antibody product. *J Pharm Sci.* (2017) 106:511–20. 10.1016/j.xphs.2016.09.028 27832839 PMC5237601

[39] Zhang L Shi S Antochshuk V. Closing the gap: counting and sizing of particles across submicron range by flow cytometry in therapeutic protein products. *J Pharm Sci.* (2017) 106:3215–21. 10.1016/j.xphs.2017.06.007 28625725

[40] Maruno T Watanabe H Yoneda S Uchihashi T Adachi S Arai K Sweeping of adsorbed therapeutic protein on prefillable syringes promotes micron aggregate generation. *J Pharm Sci.* (2018) 107:1521–9. 10.1016/j.xphs.2018.01.021 29421215

[41] Werner BP Winter G. Expanding bedside filtration—a powerful tool to protect patients from protein aggregates. *J Pharm Sci.* (2018) 107:2775–88. 10.1016/j.xphs.2018.07.022 30059660

[42] Fathallah AM Bankert RB Balu-Iyer SV. Immunogenicity of subcutaneously administered therapeutic proteins–a mechanistic perspective. *AAPS J.* (2013) 15:897–900. 10.1208/s12248-013-9510-6 23856740 PMC3787214

[43] Fathallah AM Chiang M Mishra A Kumar S Xue L Middaugh CR The effect of small oligomeric protein aggregates on the immunogenicity of intravenous and subcutaneous administered antibodies. *J Pharm Sci.* (2015) 104:3691–702. 10.1002/jps.24592 26228094

[44] Matzinger P. Tolerance, danger, and the extended family. *Annu Rev Immunol.* (1994) 12:991–1045. 8011301 10.1146/annurev.iy.12.040194.005015

[45] Fadeel B. Clear and present danger? Engineered nanoparticles and the immune system. *Swiss Med Wkly.* (2012) 142:w13609. 10.4414/smw.2012.13609 22736064

[46] Pallardy MJ Turbica I Biola-Vidamment A. Why the immune system should be concerned by nanomaterials? *Front Immunol.* (2017) 8:544. 10.3389/fimmu.2017.00544 28555135 PMC5431153

[47] Joubert MK Hokom M Eakin C Zhou L Deshpande M Baker MP Highly aggregated antibody therapeutics can enhance the *in Vitro* innate and late-stage T-cell immune responses. *J Biol Chem.* (2012) 287:25266–79. 10.1074/jbc.M111.330902 22584577 PMC3408134

[48] Telikepalli S Shinogle HE Thapa PS Kim JH Deshpande M Jawa V Physical characterization and *In Vitro* biological impact of highly aggregated antibodies separated into size-enriched populations by fluorescence-activated cell sorting. *J Pharm Sci.* (2015) 104:1575–91. 10.1002/jps.24379 25753756 PMC4448733

[49] Moussa EM Kotarek J Blum JS Marszal E Topp EM. Physical characterization and innate immunogenicity of aggregated intravenous immunoglobulin (IGIV) in an *In Vitro* cell-based model. *Pharm Res.* (2016) 33:1736–51. 10.1007/s11095-016-1914-4 27037576

[50] Polumuri SK Haile LA Ireland DDC Verthelyi D. Aggregates of IVIG or Avastin, but not HSA, modify the response to model innate immune response modulating impurities. *Sci Rep.* (2018) 8:11477 10.1038/s41598-018-29850-4 PMC6068171 30065306

[51] Tada M Aoyama M Ishii-Watabe A. Fcγ receptor activation by human monoclonal antibody aggregates. *J Pharm Sci.* (2020) 109:576–83. 10.1016/j.xphs.2019.10.046 31676270

[52] Miller L Weissmüller S Ringler E Crauwels P van Zandbergen G Seitz R Danger signal-dependent activation of human dendritic cells by plasma-derived factor VIII products. *Thromb Haemost.* (2015) 114:268–76. 10.1160/TH14-09-0789 25947149

[53] Gallais Y Szely N Legrand F-X Leroy A Pallardy M Turbica I. Effect of growth hormone and IgG aggregates on dendritic cells activation and T-cells polarization. *Immunol Cell Biol.* (2017) 95:306–15. 10.1038/icb.2016.100 27713394

[54] Ahmadi M Bryson CJ Cloake EA Welch K Filipe V Romeijn S Small amounts of sub-visible aggregates enhance the immunogenic potential of monoclonal antibody therapeutics. *Pharm Res.* (2015) 32:1383–94. 10.1007/s11095-014-1541-x 25319104

[55] Morgan H Tseng S-Y Gallais Y Leineweber M Buchmann P Riccardi S Evaluation of *in vitro* assays to assess the modulation of dendritic cells functions by therapeutic antibodies and aggregates. *Front Immunol.* (2019) 10:601. 10.3389/fimmu.2019.00601 31001248 PMC6455063

[56] Rombach-Riegraf V Karle AC Wolf B Sordé L Koepke S Gottlieb S Aggregation of human recombinant monoclonal antibodies influences the capacity of dendritic cells to stimulate adaptive T-cell responses *In Vitro*. *PLoS One.* (2014) 9:e86322. 10.1371/journal.pone.0086322 24466023 PMC3897673

[57] Joubert MK Deshpande M Yang J Reynolds H Bryson C Fogg M Use of *In Vitro* assays to assess immunogenicity risk of antibody-based biotherapeutics. *PLoS One.* (2016) 11:e0159328. 10.1371/journal.pone.0159328 27494246 PMC4975389

[58] Schultz HS Reedtz-Runge SL Bäckström BT Lamberth K Pedersen CR Kvarnhammar AM Quantitative analysis of the CD4+ T cell response to therapeutic antibodies in healthy donors using a novel T cell:PBMC assay. *PLoS One.* (2017) 12:e0178544. 10.1371/journal.pone.0178544 28562666 PMC5451071

[59] Chen L Flies DB. Molecular mechanisms of T cell co-stimulation and co-inhibition. *Nat Rev Immunol.* (2013) 13:227–42. 10.1038/nri3405 23470321 PMC3786574

[60] Land WG. Antigen in the presence of DAMPs induces immunostimulatory dendritic cells to promote destructive adaptive immune responses. In Land WG editor. *Damage-Associated Molecular Patterns in Human Diseases: Injury-Induced Innate Immune Responses.* (Vol. 1), Cham: Springer International Publishing (2018).

[61] Blanco P Palucka AK Pascual V Banchereau J. Dendritic cells and cytokines in human inflammatory and autoimmune diseases. *Cytokine Growth Factor Rev.* (2008) 19:41–52. 10.1016/j.cytogfr.2007.10.004 18258476 PMC2413068

[62] Rubic-Schneider T Kuwana M Christen B Aßenmacher M Hainzl O Zimmermann F T-cell assays confirm immunogenicity of tungsten-induced erythropoietin aggregates associated with pure red cell aplasia. *Blood Adv.* (2017) 1:367–79. 10.1182/bloodadvances.2016001842 29296951 PMC5738985

[63] Delluc S Ravot G Maillere B. Quantitative analysis of the CD4 T-cell repertoire specific to therapeutic antibodies in healthy donors. *FASEB J.* (2011) 25:2040–8. 10.1096/fj.10-173872 21368101

[64] Hamze M Meunier S Karle A Gdoura A Goudet A Szely N Characterization of CD4 T cell epitopes of infliximab and rituximab identified from healthy donors. *Front Immunol.* (2017) 8:500. 10.3389/fimmu.2017.00500 28529511 PMC5418239

[65] Meunier S Hamze M Karle A de Bourayne M Gdoura A Spindeldreher S Impact of human sequences in variable domains of therapeutic antibodies on the location of CD4 T-cell epitopes. *Cell Mol Immunol.* (2019). 10.1038/s41423-019-0304-3 (Epub ahead of print) PMC7264247 31659246

[66] Spindeldreher S Karle A Correia E Tenon M Gottlieb S Huber T T cell epitope mapping of secukinumab and ixekizumab in healthy donors. *MAbs.* (2020) 12:1707418. 10.1080/19420862.2019.1707418 31924123 PMC8648323

[67] Couceiro JR Gallardo R De Smet F De Baets G Baatsen P Annaert W Sequence-dependent internalization of aggregating peptides. *J Biol Chem.* (2015) 290:242–58. 10.1074/jbc.M114.586636 25391649 PMC4281728

[68] Eyes TJ Austerberry JI Dearman RJ Johannissen LO Kimber I Smith N Identification of B cell epitopes enhanced by protein unfolding and aggregation. *Mol Immunol.* (2019) 105:181–9. 10.1016/j.molimm.2018.11.020 30550980 PMC6344229

[69] Kijanka G Bee JS Schenerman MA Korman SA Wu Y Slütter B Monoclonal antibody dimers induced by low pH, heat, or light exposure are not immunogenic upon subcutaneous administration in a mouse model. *J Pharm Sci.* (2019) 109:730–8. 10.1016/j.xphs.2019.04.021 31029572

[70] Kumar S Mitchell MA Rup B Singh SK. Relationship between potential aggregation-prone regions and HLA-DR-binding T-cell immune epitopes: implications for rational design of novel and follow-on therapeutic antibodies. *J Pharm Sci.* (2012) 101:2686–701. 10.1002/jps.23169 22619033

